# Supplementary material for: The Interrelationship of Benefit Finding, Demoralization, and Stigma among Patients with Parkinson’s Disease and Their Caregivers
Source: Healthcare (Basel). 2024 Apr 23;12(9):878. doi: 10.3390/healthcare12090878 (PMC11083473; doi:10.3390/healthcare12090878)
Supplement: Supplementary file 1 [file healthcare-12-00878-s001.zip › healthcare-2931541-supplementary.pdf]

Supplemental Tables

Table S1 Demographic and clinical characteristics of depressive and non-depressive PD patients (N=120)

	**Depressive N (%), N=21**	**Non-depressive N (%), N=99**	**Total N (%), N=120**	**t/χ^2**	***p***
Gender				0.73	0.39
Male	14(66.7)	56(56.6)	70(58.3)		
Female	7(33.3)	43(43.4)	50(41.7)		
Age, years mean (s.d.)	61.71±8.63	67.83±8.09	66.76±8.48	-3.11	0.002
Age of onset	53.52±11.01	59.32±10.80	58.29±11.02	-2.22	0.028
Duration of PD (years)	8.19±6.19	8.48±7.47	8.43±7.24	-0.17	0.87
Years of education	12.38±4.44	11.03±4.46	11.27±4.47	1.26	0.21
Education				0.35	0.55
Less than 12 years	6(28.6)	35(35.4)	41(34.2)		
More than or equal to 12 years	15(71.4)	64(64.6)	79(65.8)		
Marital Status				3.64	0.06
Unmarried	5(23.8)	9(9.1)	14(11.7)		
Married	16(76.2)	90(90.9)	106(88.3)		
Unemployed	15(71.4)	81(81.8)	96(80.0)	1.17	0.28
Comorbid with other diseases	11(52.4)	56(56.6)	67(55.8)	0.12	0.73
Suicide history	1(4.8)	1(1.0)	2(1.7)	1.48	0.22
Anxiolytics/Hypnotics use	13(61.9)	30(30.3)	43(35.8)	7.53	0.006
Family psychiatric history				0.43	0.51
No psychiatric history	21(100.0)	97(98.0)	118(98.3)		
Depressive disorder	0	2(2.0)	2(1.7)		
NPRS	4.71±2.53	3.17±2.72	3.44±2.74	2.39	0.018
UPDRS total scores	41.05±20.06	35.58±15.86	36.78±16.91	1.28	0.20
H&Y staging	2.40±0.82	2.18±0.59	2.23±0.65	1.10	0.28
TDQ	17.00±6.36	5.85±4.43	7.80±6.41	9.65	<0.001
BFS total scores	63.52±17.28	71.58±12.69	70.17±13.87	-2.47	0.015
Acceptance	9.14±2.69	11.05±2.08	10.72±2.31	-3.62	<0.001
Family Relations	6.86±2.33	7.72±1.76	7.57±1.89	-1.60	0.12
World View	9.95±3.43	10.74±3.34	10.60±3.36	-0.97	0.33
Personal Growth	19.71±6.25	22.36±5.68	21.90±5.85	-1.91	0.06
Social Relations	8.38±4.20	9.01±3.79	8.90±3.86	-0.68	0.50
Health Behavior	9.48±2.79	10.70±2.57	10.48±2.64	-1.95	0.054
DS total scores	30.81±12.34	25.98±8.94	26.83±9.74	1.70	0.10
Loss of meaning	5.33±2.06	4.84±1.96	4.93±1.98	1.04	0.30
Dysphoria	7.33±4.05	5.34±1.97	5.69±2.56	2.20	0.039
Disheartenment	6.33±3.54	5.14±2.17	5.35±2.49	1.49	0.15
Helplessness	5.24±2.83	4.48±2.52	4.62±2.58	1.22	0.23
Sense of failure	5.19±1.60	4.94±1.83	4.98±1.79	0.58	0.56
EMIC total scores	8.86(0-27)	2.48(0-21)	3.60(0-27)	3.39	0.003

Note: Category data are presented as n (%), continuous variables are presented as score± SD.

Abbreviations: PD= Parkinson's disease; NPRS= Numerical Pain Rating Scale; UPDRS= Unified Parkinson's Disease Rating Scale; H&Y staging=Hoehn and Yahr staging; TDQ=Taiwanese Depression Questionnaire; BFS= Benefit Finding Scale; DS= Demoralization Scale; EMIC= Stigma Subscale of Explanatory Model Interview Catalogue

Table S2 Demographic and clinical characteristics of depressive and non-depressive caregivers (N=120)

	**Depressive N (%), N=16**	**Non-depressive N (%), N=104**	**Total N (%), N=120**	**t/χ^2**	***P***
Gender (%)				0.59	0.44
Male	5(31.3)	43(41.3)	48(40.0)		
Female	11(68.8)	61(58.7)	72(60.0)		
Age, years mean (s.d.)	60.81±12.56	60.81±12.82	60.81±12.73	0.001	1.00
Duration of caring (years)	8.50±5.80	8.39±7.32	8.40±7.12	0.06	0.95
Years of education	12.50±5.10	12.29±4.00	12.32±4.14	0.19	0.85
Education				0.03	0.87
Less than 12 years	4(25.0)	24(23.1)	28(23.3)		
More than or equal to 12 years	12(75.0)	80(76.9)	92(76.7)		
Marital Status				0.53	0.47
Unmarried	1(6.3)	13(12.5)	14(11.7)		
Married	15(93.8)	91(87.5)	106(88.3)		
Unemployment	9(56.3)	66(63.5)	75(62.5)	0.31	0.58
Comorbid with other diseases	9(56.3)	51(49.0)	60(50.0)	0.29	0.59
Suicide history	1(6.3)	1(1.0)	2(1.7)	2.37	0.12
Anxiolytics/Hypnotics use	6(37.5)	17(16.3)	23(19.2)	4.01	0.045
Family psychiatric history				0.16	0.69
No psychiatric history	16(100.0)	103(99.0)	119(99.2)		
Depressive disorder	0	1(1.0)	1(0.8)		
Family suicide history	0	2(1.9)	2(1.7)	0.31	0.58
NPRS	3.37(0-7)	2.36(0-10)	2.49(0-10)	1.63	0.11
TDQ	15.81(0-24)	3.17(0-17)	4.86(0-24)	8.49	<0.001
BFS total scores	64.50±10.85	74.34±13.46	73.02±13.52	-2.78	0.006
Acceptance	10.50±2.00	11.03±2.61	10.96±2.54	-0.77	0.44
Family Relations	6.13±1.75	7.60±1.64	7.40±1.72	-3.31	0.001
World View	10.19±3.29	11.82±3.35	11.60±3.37	-1.82	0.07
Personal Growth	20.50±5.77	24.34±5.31	23.83±5.51	-2.66	0.009
Social Relations	7.88±3.88	8.78±3.40	8.66±3.47	-0.97	0.33
Health Behavior	9.31±2.98	10.78±2.98	10.58±3.01	-1.84	0.07
DS total scores	32.50±14.24	24.36±9.45	25.44±10.52	2.21	0.041
Loss of meaning	4.75±3.75	4.66±2.28	4.68±2,51	0.09	0.93
Dysphoria	7.94±4.07	5.12±2.33	5.49±2.78	2.70	0.015
Disheartenment	7.44±4.55	5.17±2.52	5.48±2.95	1.95	0.07
Helplessness	5.19±2.88	3.88±1.83	4.06±2.04	1.76	0.10
Sense of failure	5.56±1.71	4.45±2.19	4.60±2.16	1.94	0.06
EMIC total scores	5.81(0-15)	1.98(0-17)	2.49(0-17)	2.53	0.022

Note: Category data are presented as n (%), continuous variables are presented as score± SD.

Abbreviations: PD= Parkinson's disease; NPRS= Numerical Pain Rating Scale; UPDRS= Unified Parkinson's Disease Rating Scale; H&Y staging=Hoehn and Yahr staging; TDQ=Taiwanese Depression Questionnaire; BFS= Benefit Finding Scale; DS= Demoralization Scale; EMIC= Stigma Subscale of Explanatory Model Interview Catalogue
